# Kisspeptin Administration and mRNA Expression in Adult Syrian Hamsters

**DOI:** 10.3390/cells14130992

**Published:** 2025-06-29

**Authors:** Megan A. L. Hall, Peyton L. Reeder, Johnathan M. Borland, Robert L. Meisel

**Affiliations:** Department of Neuroscience, University of Minnesota, Minneapolis, MN 55455, USA; reede076@umn.edu (P.L.R.); borla040@umn.edu (J.M.B.); meisel@umn.edu (R.L.M.)

**Keywords:** kisspeptin, kisspeptin receptor, in situ hybridization, Syrian hamster, estrous cycle, CNS distribution

## Abstract

Kisspeptin (*Kiss1*) and kisspeptin 1 receptor (*Kiss1R*) are vital in regulating various functions across many species, primarily those relating to reproduction. The kisspeptin system has recently attracted clinical interest as a potential therapeutic treatment for patients with hypoactive sexual desire disorder. This study maps the distribution of *Kiss1* and *Kiss1R* mRNA in the Syrian hamster forebrain using dual-labeled RNAscope. In our study, the distributions of kisspeptin and its receptor were mapped across adult males and females on day 1 or day 2 of their estrous cycle. Conditioned place preference was used to observe the potential effect of kisspeptin on sexual reward in female hamsters. The expression of kisspeptin was greater in females than males, with the estrous cycle having no effect on expression. A comparison of these findings to those in other species revealed that the expression in Syrian hamsters was similar to that reported for other species, demonstrating the conservation of expression. Kisspeptin did not influence sexual reward in females, nor did it affect measures of their primary sexual behavior. These findings provide additional insights into the expression and function of kisspeptin across novel species and add to ongoing research in understanding how kisspeptin may influence sexual desire in animals, including humans.

## 1. Introduction

Kisspeptin is a neuroendocrine modulator that is primarily expressed in hypothalamic neurons. These neurons are part of the hypothalamic–pituitary–gonadal axis, best known for the coordinated regulation of gonadotropin-releasing hormone (GnRH) from the hypothalamus; in turn, this affects reproductive development, beginning prenatally and extending through puberty and into adulthood [1]. In this regard, kisspeptin is produced by and released from neurons in the anteroventral periventricular and arcuate nuclei, which in turn synapse on GnRH neurons [2,3], with the medial amygdala serving as an additional site of kisspeptin production [4,5,6,7,8]. In adult females, for example, these brain regions work in concert to generate a pattern of pulsatile GnRH secretion, with a corresponding pulsatile release of follicle-stimulating hormone (FSH) and luteinizing hormone (LH) from the anterior pituitary [9,10,11,12]. This pulsatile release of FSH and LH promotes the asynchronous synthesis and release of estrogens and progestins in the rodent ovary, as well as a surge in FSH and LH, leading to ovulation [13]. Over a longer period, kisspeptin is part of seasonal patterns of reproductive competence [14].

The focus on the restricted distribution of kisspeptin neurons and their relation to reproductive physiology is countered by the more widespread distribution of kisspeptin fibers and receptors within the brain [15]. For example, kisspeptin regulates metabolic activity that includes locomotion [16,17], food intake [17,18], and the metabolic energy balance [19,20,21]. Furthermore, in addition to reproductive physiology, it makes sense that kisspeptin pathways would be integral to cyclically coordinating reproductive behavior with ovulation [22,23,24].

Our interest in kisspeptin originates from its potential involvement in the motivational control of female reproductive behavior [23,25] and the potential rewarding consequences of these interactions. To address this concept, two approaches were used: The first involved mapping the forebrain distribution of kisspeptin neurons in male and female hamster brains, using the RNAscope-based in situ hybridization labeling of *Kiss1* mRNA. The distribution of the primary kisspeptin receptor (*Kiss1R*) was mapped through the in situ hybridization of *Kiss1R* in the same tissue sections. These mapping studies were important, as a variety of species-specific differences have been reported in relation to the distribution of kisspeptin neurons and fibers [26,27], with limited assessments of kisspeptin neuron distributions in Syrian hamsters [26] and no comparable studies for kisspeptin receptor expression. Female Syrian hamsters readily display sexual receptivity to males on day one of their four-day estrous cycle, with females being highly aggressive toward the males on the other three days [28]. With this in mind, the distribution patterns of kisspeptin peptide and kisspeptin receptors were compared between males and females and within females between the day on which they were sexually receptive to males and the following day, when the females responded to the males aggressively. Finally, we tested the potential rewarding properties of kisspeptin in females using our established conditioned place preference paradigm [29,30,31,32].

## 2. Materials and Methods

### 2.1. Subjects

Adult female and male Syrian hamsters (*Mesocricetus auratus*) were purchased from Charles River Laboratories (Wilmington, MA, USA) at approximately 60 days of age (130 to 160 g). Females and males were housed individually in polycarbonate cages (50.8 × 40.6 × 20.3 cm). All animals were maintained on a reversed 14 h:10 h light/dark photoperiod (lights off at 1300 h). The animal room was maintained at a controlled temperature of 22 °C, and food and water were available ad libitum. All animal procedures were carried out in accordance with the National Institutes of Health Guide for the Care and Use of Laboratory Animals (NIH Publications No. 80-23; revised 2011) and approved by the University of Minnesota Institutional Animal Care and Use Committee.

### 2.2. Cycle Tracking

The characterization of the estrous cycles was based on the external appearance of the female hamster’s vaginal secretion, as first described by Orisini [33]. On the day of behavioral estrus, the vaginal secretion has a stringy appearance, which is followed by a more copious secretion the following day [34]. The stringy appearance was labeled as occurring on day 1 of the cycle, with the more copious secretion representing day 2 [34]. These 2 days of the cycle were chosen for the analyses as representing behaviorally divergent stages: females are typically sexually responsive to a male hamster on day 1, while they will attack an intruding male on day 2 [28].

### 2.3. Histology

Subjects were injected with a pentobarbital-based euthanasia solution (Beuthanasia-D, 0.25 mL i.p., Schering, Union, NJ, USA) and sacrificed via rapid decapitation. Brains were extracted, frozen in an optimum cutting temperature compound (VWR Scientific Products, Radnor, PA, USA), and stored for later sectioning at −80 °C. The brains were cryostat-sectioned coronally at a thickness of 14 µm, and sections were mounted on Colorfrost Plus slides (Fisher Scientific, Waltham, MA, USA). Some studies in rats and mice [2,26,27,35] have provided an extensive analysis of the range of the distribution of kisspeptin and kisspeptin receptor-expressing neurons. Here, the focus was the forebrain, with tissue collected from slices containing the prefrontal cortex through to slices containing the posterior hypothalamus. Slides were stored at −80 °C until we conducted RNAscope procedure treatment.

### 2.4. RNAscope

The ACDBio RNAscope Multiplex Fluorescent V2 Assay protocol was used as described by Hall et al. [36]. Probes targeting Syrian hamster sequences used in the procedure were designed using Advanced Cell Diagnostics (Newark, CA, USA): Mau-Kiss1-C1 (mRNA encoding kisspeptin 1, *Kiss1*; GenBank accession number NM_001281568.1, target nt region: 2–657) and Mau-Kiss1R-C2 (mRNA encoding kisspeptin 1 receptor, *Kiss1R*; GenBank accession number XM_013122973.3, target nt region: 1231–2784). Each tissue section received 75 µL of a solution that contained a 50:50 ratio of probe diluent (Advanced Cell Diagnostics) and probe mixture. The probe mixture contained a 50:1 ratio of C1:C2, such that each tissue section received 37.50 µL of probe diluent, 36.75 µL of C1, and 0.75 µL of C2. Fluorescently labeled Kiss1-C1 was associated with Opal 570 nm, at a concentration of 1:750, and Kiss1R-C2 with Opal 520 nm, at a concentration of 1:750.

### 2.5. Confocal Imaging

Slides were scanned with a fluorescence microscope to identify brain regions that expressed either *Kiss1* or *Kiss1R* mRNA. Sixteen brain regions were chosen based on the findings reported in previous studies, functional sites of kisspeptin action, and visual inspections of expression within tissue by the same experimenter (MAL Hall). The brain regions were identified from low-power images based on the patterns of DAPI labeling (through the coverslip medium) and anatomical landmarks that were visible in the fluorescent images, which were then compared to plates in a Syrian hamster stereotaxic atlas [37]. Schematic diagrams from the hamster atlas were selected from the levels that corresponded to the imaged regions of interest, and these plates were overlaid with a box to illustrate the location of the mRNA labeling to be analyzed. Sections containing the regions of interest were selected from specific locations (matched across brains), based on the atlas plates; they represent a rostral–caudal distance of less than 0.5 mm.

Images were acquired using a Leica TCS SPE confocal microscope (Wetzlar, Germany) under the same scanning parameters as reported in our prior studies [36]. For each subject, images were collected from the left and right hemispheres from two tissue sections (in series), resulting in a sample of four images for each brain region. Images were collected with a 20×/0.60 advanced correction system objective with a pixel distribution of 1024 × 1024 at a frequency of 8 kHz. A dual-laser system with 488 and 532 nm wavelengths and an ultra-high dynamic PMT detector captured z-stack images at 1.5 µm per step for a maximum of 15 steps. The pinhole size was 1 airy unit (AU), and the optical zoom was 1.00×. The dimensions of the 3D images produced were 550 × 550 × 14 µm.

### 2.6. Image Data Collection

**Figure 1:**
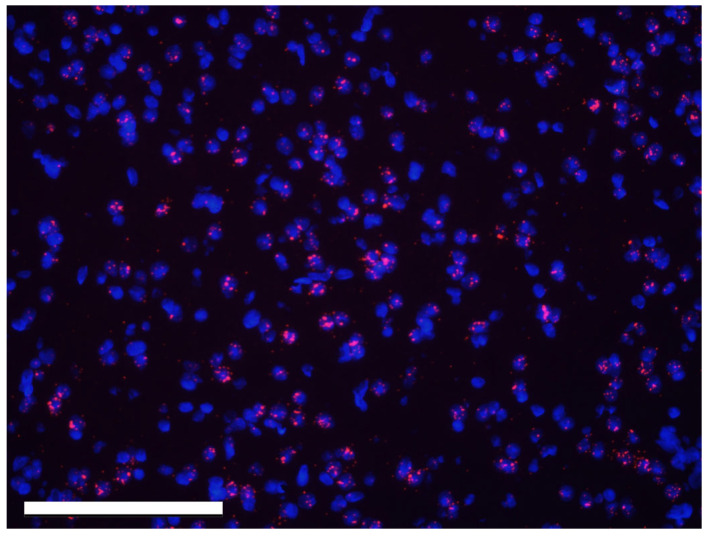
DAPI (blue) colocalization with *Kiss1R* mRNA puncta (red), as seen in Imaris prior to applying the Spots model. The scale bar is 500 µM.

**Figure 2:**
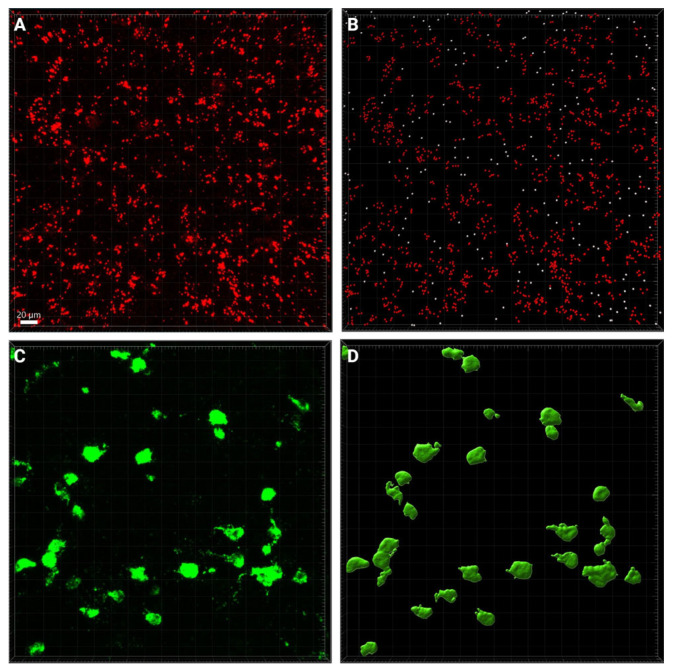
(**A**) The raw image of mRNA puncta is pictured on the left. (**B**) The Spots model on the right demonstrates the one-to-one count of puncta in red that met the parameters described, while the puncta in white did not meet the parameters, so were not counted. (**C**) The raw image of *Kiss1* mRNA clusters is pictured on the left. (**D**) The Surface model on the right illustrates the one-to-one count of mRNA clustering in cells that met the parameters described. The scale bar of 20 µM presented in (**A**) is consistent across the images.

Images were analyzed using Imaris software (Oxford Instruments, Oxfordshire, England; version 9.7.2) to investigate (1) the number of puncta of *Kiss1R* mRNA and (2) the number of cells that express *Kiss1* mRNA. The Imaris Spots feature was used to create a model of the *Kiss1R* mRNA channel. For *Kiss1R* labeling, DAPI counterstaining was used to confirm the localization of mRNA puncta to cells (Figure 1), with an estimated XY diameter set at 0.650 µm to determine the thresholds of the puncta. Next, the “quality” filter was applied, with the threshold adjusted manually to include all spots that met the diameter in each channel. Then, the “average distance to three nearest neighbors” filter was applied for all spots that passed the quality filter. This was necessary because the expression level of the receptor was too low to define labeled cells based on spot labeling itself. The number of spots that were filtered into the model was then recorded for the *Kiss1R* channel (Figure 2A,B).

The Imaris Surfaces feature was used to create a model of the *Kiss1* channel to estimate the number of cells that express *Kiss1* mRNA. The cellular threshold for absolute intensity was set with a seed point diameter of 15 µm. The “quality” filter was first applied with the threshold adjusted manually to identify a seed point for all possible cells in each channel. Second, the “area” filter was set at 19.63 µm^2^ and a “volume” filter was applied to include in the model only cells with a volume greater than 65.45 µm^3^. The number of cells that were filtered into the model was then recorded for the *Kiss1* channel. The criteria for cell surfaces excluded any potential fractured cells that may be present in the image (Figure 2C,D).

### 2.7. Hormone Treatment to Induce Sexual Receptivity

To control the timing of sexual receptivity for conditioned place preference, female hamsters (n = 7–10/group) were bilaterally ovariectomized using aseptic surgical procedures while under isoflurane anesthesia (3% in O_2_; Piramal Pharma Limited, Telangana State, India). A subcutaneous (s.c.) analgesic (Meloxicam, 1 mg/kg body weight; Fort Dodge Animal Health, Overland Park, KS, USA) was administered 30 min prior to surgery and three days after for post-operative pain management. Ovariectomized hamsters were primed with hormones weekly for lordosis behavior scoring via s.c. injections of estradiol benzoate (10 μg in 0.1 mL of cottonseed oil; Sigma-Aldrich, Burlington, MA, USA; CAS-No. 8001-29-4) at approximately 48 and 24 h prior to behavior testing and the s.c. injection of progesterone (500 μg in 0.1 mL of cottonseed oil; Sigma-Aldrich, Burlington, MA, USA; CAS-No. 8001-29-4) approximately 4 h prior to the behavior tests.

### 2.8. Conditioned Place Preference

Females were given sex behavior tests in the context of the lab’s conditioned place preference (CPP) procedure, which was designed to assess the rewarding properties of sex behavior [32]. The CPP apparatus consists of two lateral chambers (60 × 45 × 38 cm) connected by a clear central chamber (37 × 22 × 38 cm). The lateral chambers are differentiated by the color of the walls and the type of bedding on the chamber floor. One lateral chamber was gray and contained Aspen bedding (Harlan Laboratories, Indianapolis, IN, USA), while the other lateral chamber was white and contained 1/8-inch corncob bedding (Harlan Laboratories). Female hamsters underwent three different procedures in the CPP apparatus to assess sexual reward. Hamsters were first given a pre-test, during which hormone-primed female hamsters were placed in the clear central chamber and allowed to freely explore the entire apparatus for 10 min. This experience established an initial preference for one of the lateral compartments. They then received 2 weekly conditioning tests in which the hormone-primed females were given a sexual experience by pairing them with an adult male hamster in their initially non-preferred chamber for 10 min. One hour later, these females were placed alone into their initially preferred chamber for 10 min. The order in which the conditioning stimuli (i.e., a sexually experienced male versus no stimulus) are presented does not impact the formation or strength of a subsequent conditioned place preference [32]. One week after the two conditioning sessions, hormone-primed females were given a post-test, during which they were again allowed to freely explore the entire apparatus for 10 min. Subjects received 0.1 mL s.c. injections of either 1 µM or 10 µM of kisspeptin (KP; Kisspeptin-10, #048-56, Phoenix Pharmaceuticals, Burlingame, CA, USA) or vehicle (0.9% saline) 1.5 h prior to the first conditioning session of the day.

### 2.9. Behavior Scoring

Video recordings of CPP conditioning sessions were watched to measure the lordosis latency and lordosis duration for each group. The first five minutes of each video were watched and a stopwatch was used to record the times for each dataset. The stopwatch was started from the time the male subject was placed in the chamber with the female. Lordosis latency was determined by the onset to the first lordosis posture (the ventral arching of the female’s back) from the time that the male was placed into the chamber. The lordosis duration was determined according to the cumulative amount of time the female spent in lordosis after the onset of the first sign of lordosis.

### 2.10. Statistics and Analysis

Data were graphed using the GraphPad Prism software (version 8.4.3, San Diego, CA, USA), and the graphs display the mean ± standard error of the mean. Alpha (α) was set to 0.05. First, normality was assessed using the Shapiro–Wilk test. All datasets were normally distributed. A two-way analysis of variance (ANOVA) statistical test and *t*-tests were performed for the CPP, lordosis latency, and lordosis duration to identify differences among treatments (saline, 1 µM KP, and 10 µM KP) and CPP tests (pre-test versus post-test). A one-way ANOVA was performed for the kisspeptin RNAscope data to identify differences among males, day 1 females, and day 2 females. Tukey’s post hoc comparison test was used.

## 3. Results

### 3.1. Effect of Sex and the Estrous Cycle on Kisspeptin mRNA Expression

**Figure 3:**
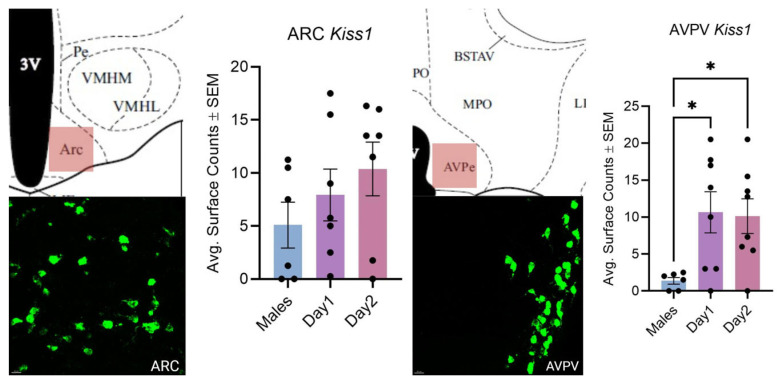
Kisspeptin mRNA expression in the ARC (**left**) and AVPV (**right**) for males and day 1 as well as day 2 females. The schematic in the top left of each set illustrates the region imaged. The fluorescent image in the bottom left of each set is kisspeptin expression in the ARC or AVPV taken on the confocal apparatus and used for Imaris. For the bar graph, the bars indicate the average number of cells that express kisspeptin mRNA across males, day 1 females, and day 2 females. Neither sex nor the estrous cycle had any significant effects on the ARC. There is a significant difference in expression between day 1 females (*p* < 0.05) and day 2 females (*p* < 0.05) when compared to males in the AVPV (* indicates *p* < 0.05). The scale bar is 20 µM.

In the ARC, there were no significant differences in kisspeptin mRNA expression between males, estrous females, and diestrous females (Figure 3; F (2, 17) = 1.160, *p* > 0.05). While not significantly different, there was a positive trend in diestrous females having a greater average number of cells that expressed kisspeptin mRNA compared to both day 1 females and day 1 males.

There were significant differences for kisspeptin mRNA expression in the AVPV (Figure 3, F (2, 19) = 4.575, *p* < 0.05). Day 1 and day 2 females had significantly higher kisspeptin mRNA expression when compared to males; however, there were no significant differences in expression between day 1 and day 2 females.

**Figure 4:**
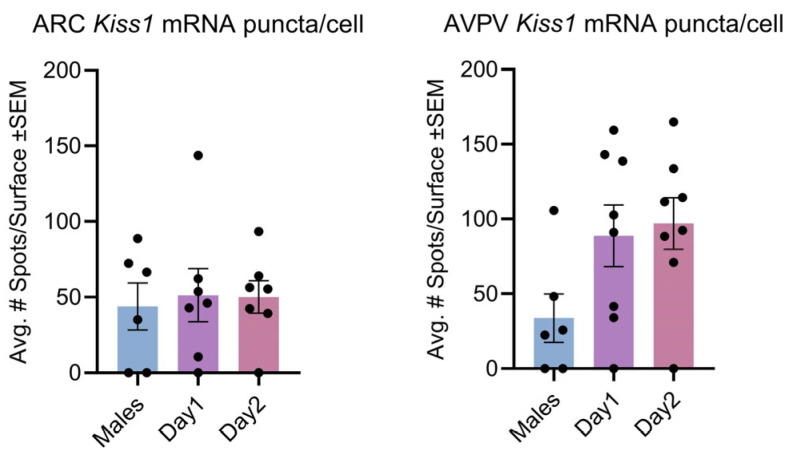
Kisspeptin mRNA puncta per cell for the ARC and AVPV. Cycle had no significant effect on the number of puncta expressed per cell in the ARC (F (2, 17) = 0.06910, *p* > 0.05), nor in the AVPV (F (2, 19) = 3.070, *p* > 0.05).

There were no significant differences in the amount of kisspeptin mRNA puncta per cell as a result of the cycle (Figure 4). This was calculated to determine whether the amount of expression per cell may differ, while the number of cells that express kisspeptin did not differ.

### 3.2. Effect of Estrous Cycle on Kisspeptin Receptor mRNA Expression

The kisspeptin receptor mRNA expression was present throughout the entire range of forebrain tissue. The regions that were analyzed for the receptor mRNA were chosen based on functions related to sex behaviors and reward-seeking behaviors.

**Figure 5:**
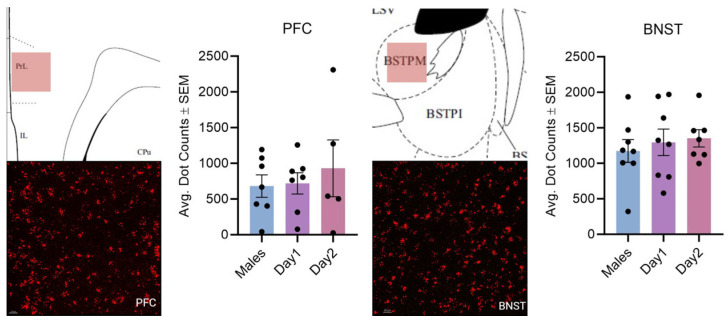
Kisspeptin receptor mRNA expression in the PFC and BNST for males and day 1 as well as day 2 females. The schematic in the top left illustrates the imaged region. The image in the bottom left is the expression of kisspeptin receptor 1 mRNA. In the graph, the bars indicate the average number of kisspeptin mRNA puncta for the region per group. For the PFC: F (2, 16) = 0.3112, *p* > 0.05; BNST: F (2, 20) = 0.3143, *p* > 0.05. The scale bar is 20 µM.

**Figure 6:**
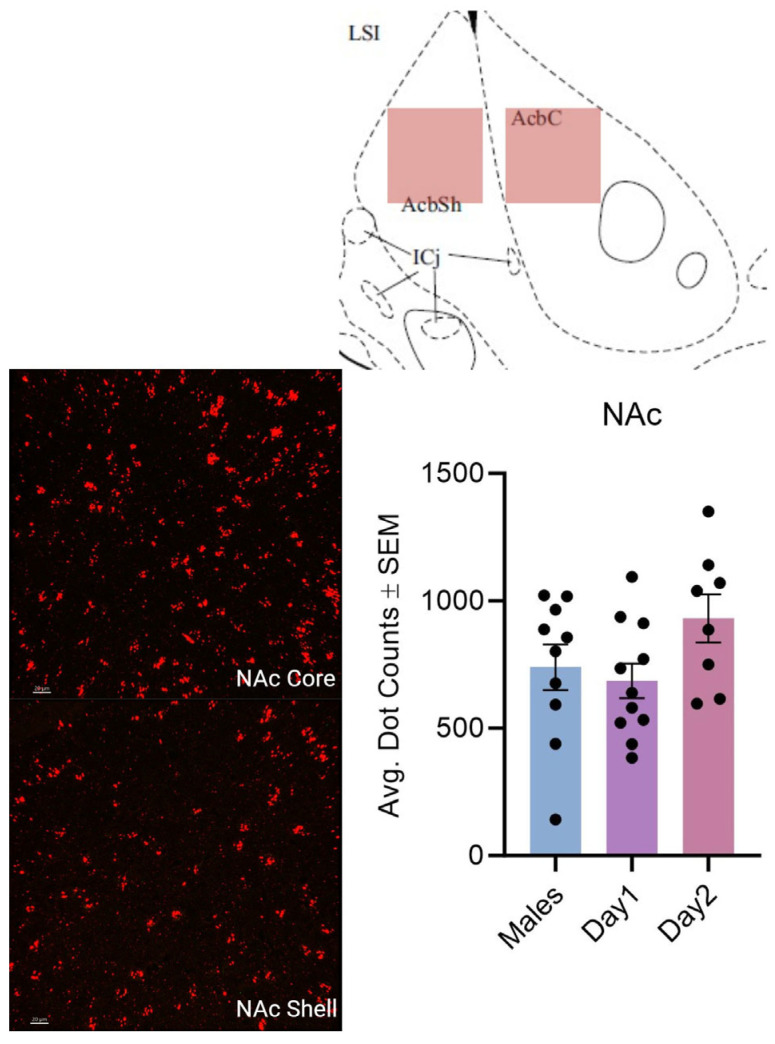
Kisspeptin receptor mRNA expression in the NAc core and shell for males and day 1 as well as day 2 females. The schematic at the top illustrates the imaged region. The images to the left are the expression of kisspeptin receptor 1 mRNA. In the graph, the bars indicate the average number of kisspeptin mRNA puncta for the region (shell and core combined) per group. For the NAc, F (2, 26) = 2.220, *p* > 0.05. The scale bar is 20 µM.

**Figure 7:**
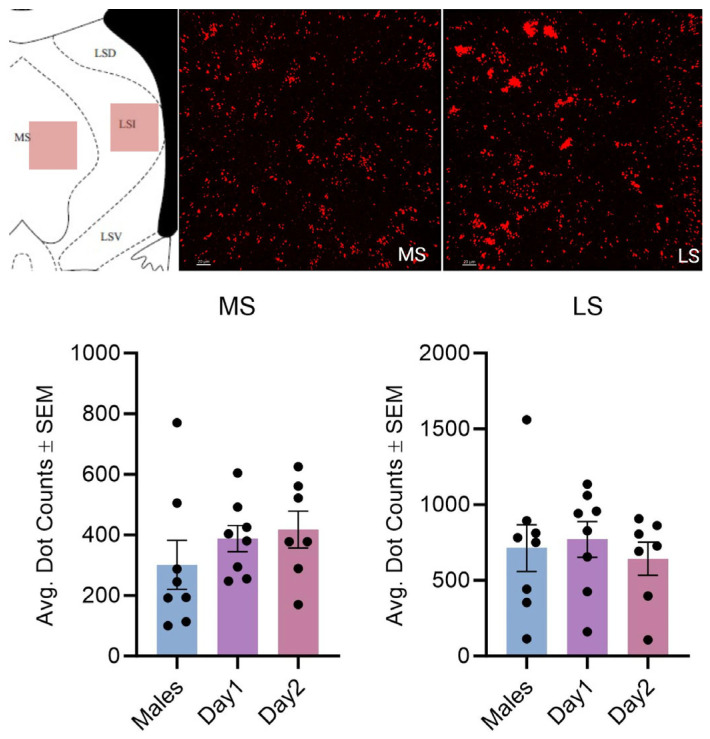
Kisspeptin receptor mRNA expression in the MS and LS for males and day 1 as well as day 2 females. The schematic in the top left illustrates the regions imaged. The images in the top right show the expression of kisspeptin receptor 1 mRNA. In the graph, the bars indicate the average number of kisspeptin mRNA puncta for the region per group. For the MS, F (2, 20) = 0.8977, *p* > 0.05; LS: F (2, 20) = 0.2325, *p* > 0.05. The scale bar is 20 µM.

**Figure 8:**
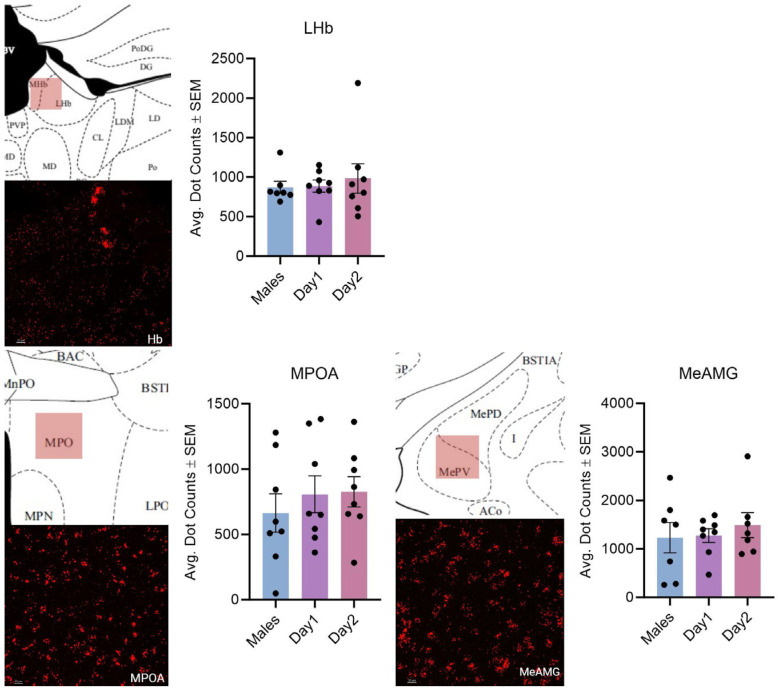
Kisspeptin receptor mRNA expression in the LHb, MPOA, and MeAMG for males and day 1 as well as day 2 females. The schematic in the top left of each set illustrates the imaged region. The image in the bottom left is the expression of kisspeptin receptor 1 mRNA. In the graph, the bars indicate the average number of kisspeptin mRNA puncta for the region per group. For the LHb, F (2, 20) = 0.2307, *p* > 0.05; MPOA: F (2, 21) = 0.4299, *p* > 0.05; and MeAMG: F (2, 19) = 0.3197, *p* > 0.05. The scale bar is 20 µM.

**Figure 9:**
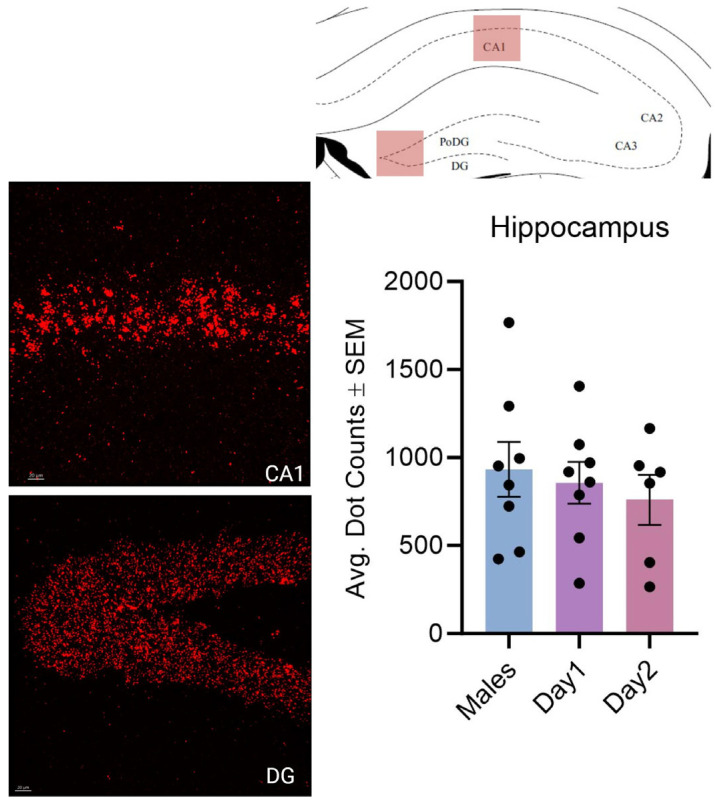
Kisspeptin receptor mRNA expression in the CA1 and DG of the hippocampus combined for males and day 1 as well as day 2 females. The schematic at the top illustrates the region imaged. The images to the left show the expression of kisspeptin receptor 1 mRNA. In the graph, the bars indicate the average number of kisspeptin mRNA puncta for the region (CA1 and DG combined) per group. For the hippocampus, F (2, 19) = 0.3528, *p* > 0.05. The scale bar is 20 µM.

**Figure 10:**
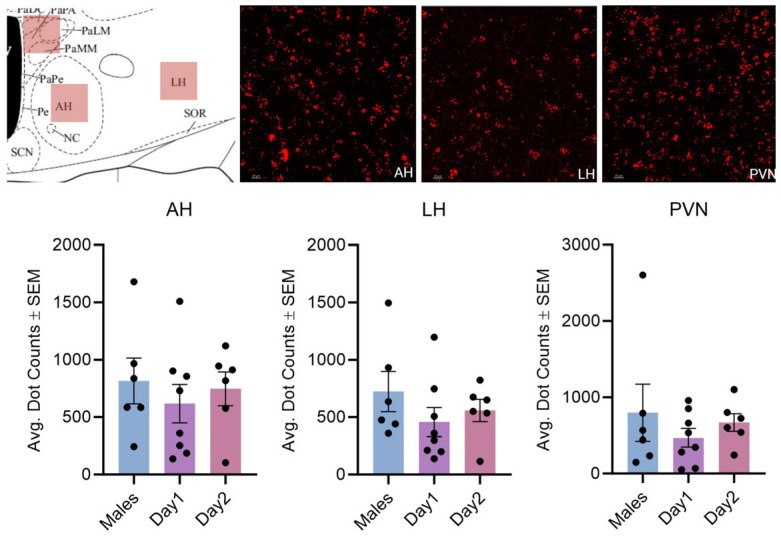
Kisspeptin receptor mRNA expression in the AH, LH, and PVN for males and day 1 as well as day 2 females. The schematic in the top left illustrates the region imaged. The images in the top right show the expression of kisspeptin receptor 1 mRNA. In the graph, the bars indicate the average number of kisspeptin mRNA puncta for the region per group. For the AH, F (2, 17) = 0.3615, *p* > 0.05; LH: F (2, 17) = 0.9796, *p* > 0.05; and PVN: F (2, 17) = 0.6025, *p* > 0.05. The scale bar is 20 µM.

**Figure 11:**
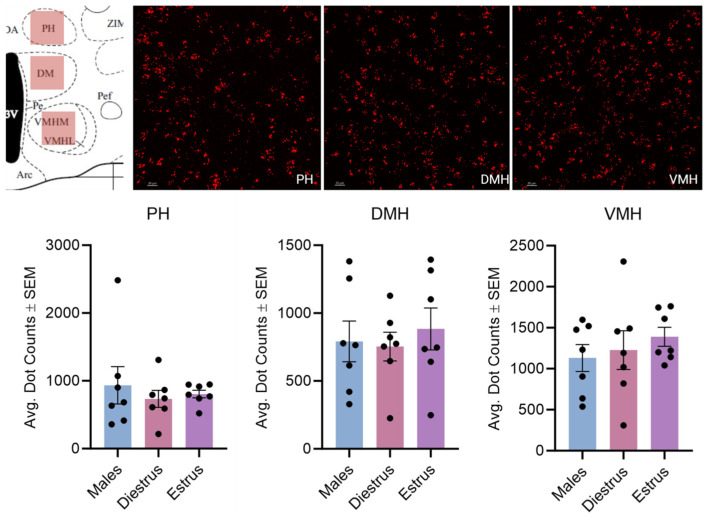
Kisspeptin receptor mRNA expression in the PH, DMH, and VMH for males and day 1 as well as day 2 females. The schematic in the top left illustrates the region imaged. The images in the top right show the expression of kisspeptin receptor 1 mRNA. In the graph, the bars indicate the average number of kisspeptin mRNA puncta for the region per group. For the PH, F (2, 18) = 0.3324, *p* > 0.05; DMH: F (2, 18) = 0.2315, *p* > 0.05; and VMH: F (2, 18) = 0.5356, *p* > 0.05. The scale bar is 20 µM.

Overall, the estrous cycle had no significant effects on kisspeptin receptor mRNA in any of the regions analyzed (Figure 5, Figure 6, Figure 7, Figure 8, Figure 9, Figure 10 and Figure 11).

### 3.3. Effect of Kisspeptin on Lordosis

**Figure 12:**
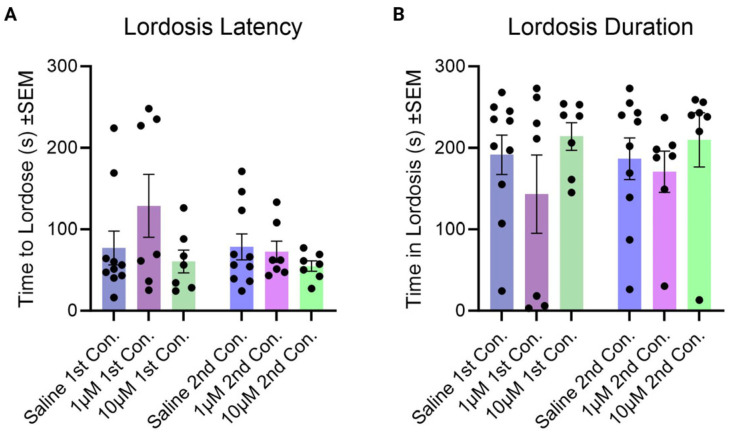
(**A**) Lordosis latency and (**B**) lordosis duration. Bars indicate the average time it took for subjects to enter lordosis for each group. Blue bars indicate subjects that received saline. Purple bars indicate subjects that received 1 µM of kisspeptin-10. Green bars indicate subjects that received 10 µM of kisspeptin-10. The darker-shaded bars indicate the first conditioning session, and the lighter-colored bars indicate the second conditioning session.

The lordosis behavior of the females in the two conditioning sessions was analyzed, and we observed how long it took them to enter lordosis (lordosis latency) and, cumulatively, how long they stayed in lordosis (lordosis duration) for the first five minutes of each test. Neither treatment nor number of tests had a significant effect on lordosis latency (Figure 12A; number of tests x treatment interaction, F (2, 21) = 0.9649, *p* > 0.05; test factor, F (1, 21) = 1.218, *p* > 0.05; treatment factor, F (2, 21) = 2.305, *p* > 0.05) or lordosis duration (Figure 12B; number of tests x treatment interaction, F (2, 21) = 0.1569, *p* > 0.05; test factor, F (1, 21) = 0.05330, *p* > 0.05; treatment factor, F (2, 21) = 1.824, *p* > 0.05).

### 3.4. Effect of Kisspeptin Treatment and Tests on Conditioned Place Preference

**Figure 13:**
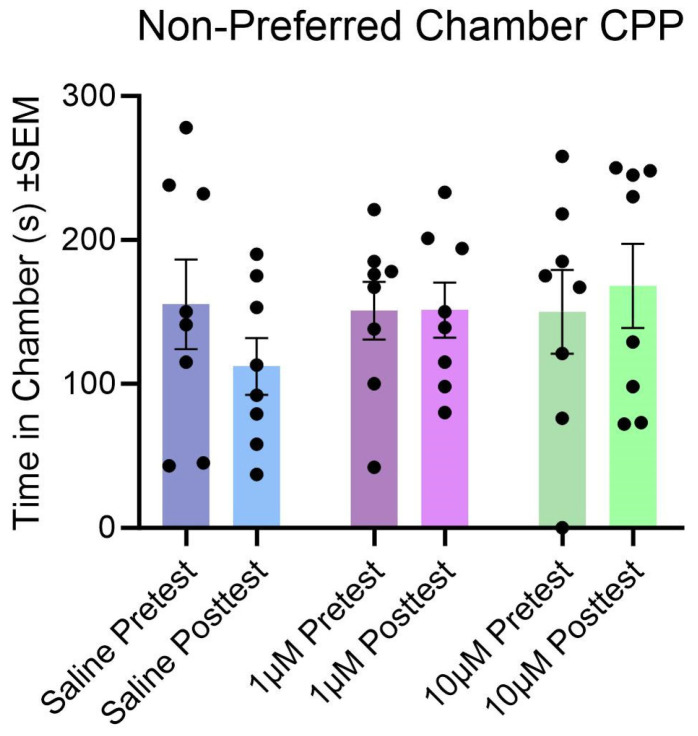
Pre- and post-test comparison of time spent in the non-preferred chamber after two sessions of CPP. Blue bars indicate subjects that received saline. Purple bars indicate subjects that received 1 µM of kisspeptin-10. Green bars indicate subjects that received 10 µM of kisspeptin-10. The darker bars indicate the pre-test session, and the lighter bars indicate the post-test session.

Within each treatment group, we analyzed the changes in the time the animals spent in their non-preferred chamber in their post-test when compared to the pre-test. It was hypothesized that there would be an increase in the time spent in the post-test compared to their pre-test. Females did not significantly increase the time they spent in the non-preferred chamber when comparing the pre- and post-tests (Figure 13; F (1, 21) = 0.2279, *p* > 0.05).

The effect of the dose treatment across the pre-test and post-test was also analyzed. There were no significant differences between saline, low-dose KP, and high-dose KP in the pre-test or post-test (F (2, 21) = 0.4064, *p* > 0.05). There was also no significant interaction between tests and treatments for the groups (F (2, 21) = 1.120, *p* > 0.05).

## 4. Discussion

The primary goal of this study was to analyze the effect of sex and the females’ reproductive cycle on the expression of kisspeptin and kisspeptin 1 receptor across the forebrains of Syrian hamsters, as well as examining the potential effects of kisspeptin on reward-seeking and sex behaviors in females.

Kisspeptin and kisspeptin 1 receptor expression have many similarities and differences across species. The localization of kisspeptin to a restricted set of basal forebrain regions is common to all species [38]. In rodent species, two of these rostral areas are the AVPV and surrounding areas, collectively coined the rostral periventricular area of the third ventricle (RP3V), as well as the ARC nucleus. For other species, kisspeptin is also expressed in the MeAMG in mice [4] and in the MPOA in mice, sheep, and primates [26,39,40]. These regions work in concert to modulate gonadotrophin release and, subsequently, ovarian cyclicity [41]. The RNAscope analyses of kisspeptin expression are consistent with the results of previous rodent studies, with the kisspeptin mRNA expression in hamsters largely contained to the AVPV and ARC [26,42,43]. Furthermore, in the AVPV, females on both day 1 and day 2 of their cycle had significantly greater kisspeptin mRNA expression than the males did. In the ARC, there was a trend towards day 2 females having a greater number of cells that expressed kisspeptin mRNA compared to both day 1 females and males. Again, our findings align with those previously described in the literature [44,45].

In contrast to kisspeptin protein, kisspeptin 1 receptor expression was much more abundant across the central nervous system among the species studied [35,46]. For example, Zhang et al. [47] used RNAscope to map the distribution of kisspeptin receptor expression throughout the brain in mice. Unsurprisingly, sagittal views of this expression show regional differences in the intensity of receptor labeling, but the key finding is the universal localization of the receptor from the olfactory bulbs to the cerebellum and brainstem. The labeling of Syrian hamster forebrain regions expressing the receptor using RNAscope confirms the ubiquitous expression of the kisspeptin 1 receptor, with regional variations in intensity. Numerically, the MeAMG stood out as having the highest expression levels for the receptor mRNA puncta in males and females on day 2 of their estrous cycle. Further, females on day 1 of their cycle (corresponding to the timing of sexual receptivity) had the highest receptor mRNA puncta expression in the VMH, a region critical for the hormonal activation of female sexual behavior. The variability present in the mRNA expression levels across subjects may require increasing the number of subjects in future studies. What is unclear from these receptor analyses is the origin of the kisspeptin innervation of these regions of receptor expression, given the restricted distribution of the kisspeptin expression itself—a critical point raised by Bakker in an insightful review [25]. One possible explanation for this is that, as more-sophisticated techniques are utilized, more cellular regions expressing kisspeptin will be discovered [48].

The inability of kisspeptin to potentiate sexual reward in our study marks a contrast to the positive effects of kisspeptin on CPP demonstrated in rats [49]. It is certainly possible that there are species-specific differences in the ability of kisspeptin to promote sexual reward. On the other hand, there are several procedural differences between the current study in Syrian hamsters and the Bedos study of rats [49]. One difference is the duration of sex behavior engaged in during the CPP procedure. The hamsters were given 10 min of exposure to males during the conditioning sessions, whereas the rats received one hour of sexual interaction. Another difference is the dose of kisspeptin-10 the subjects received. The doses of kisspeptin-10 were chosen based on their relative ability to stimulate LH release in Siberian hamsters [50]. Our doses of kisspeptin that were ineffective in producing CPP were several orders of magnitude higher than the effective doses (7 and 14 nmol) used in rats [49]. Superficially, we think of higher doses as being more potent; however, there are examples in which other peptides have inverted-U dose–response curves [51,52]. It is possible that the high doses of kisspeptin were beyond the effective range for producing a CPP response, and variability in subject responses may occur as a result.

As noted, our interest in kisspeptin originates from its possible use as a therapeutic agent for sexual dysfunction. Hypoactive sexual desire disorder (HSDD) is an absence or decrease in sexual desire in both women and men [53,54]. It is the most prevalent form of a low sex drive, with the premenopausal prevalence ranging from 12.2% in women aged 18–24 years old to 33.4% in women aged 40–44 years old [25]. Women who are diagnosed with HSDD are found to have hyperactivity in cortical regions, which act to inhibit limbic regions, resulting in interference with sex drive. Indeed, recent studies have turned to kisspeptin administration as a potential treatment for women and men with HSDD [25].

Thurston and colleagues [53] found that women who received intravenous injections of kisspeptin were found to have decreased activity in the left inferior gyri, middle front gyri, and temporoparietal junction. These regions are involved in inhibitory control, internal monologs, perception of others, and self-evaluation. Increased activation of regions such as the hippocampus, posterior cingulate cortex, and supramarginal and post-central gyrus was also present, and these areas are involved in increasing sexual desire and romantic feelings [53]. The deactivation and activation of these regions, respectively, by kisspeptin could allow women to think less negatively of themselves or others and focus more on their sexual desires, a process that may apply equally to men [54,55]. Collectively, these studies advance our understanding of how kisspeptin may affect patients with HSDD; overall, they demonstrate benefits in terms of increasing sexual desire and arousal.

This study both confirms and extends the neural localization of kisspeptin and its cognate receptor, as reported previously in Syrian hamsters and other rodent species. Because sexual behavior in people has complex underpinnings, utilizing a diverse set of model species that includes varied preclinical behavioral tests and endpoints becomes the most fruitful way to develop therapeutic approaches to treating sexual dysfunctions. To our knowledge, this study is the first to analyze the mRNA expression of the kisspeptin system in our validated Syrian hamster model for sexual motivation and reward [56]. The results localizing kisspeptin neurons in the hypothalamus, as well as the diffuse distribution of kisspeptin receptors throughout the forebrain, support the continued use of Syrian hamsters in kisspeptin research. The mismatch between the localization of the kisspeptin-expressing neurons and the diffuse localization of receptors means that other kisspeptin neuronal cell groups must exist outside the range of the present analyses. This will be a focus of our future research. Furthermore, this CPP study does not support the role of sexual reward in hamsters; we therefore require a broader range of endpoints to comprehensively evaluate kisspeptin’s role in female sexual motivation and desire. Collectively, such studies will represent a rational basis for preclinical approaches to developing kisspeptin as a target for treating HSSD in women, as well as discovering other potential therapeutics for the treatment of sexual dysfunctions in people [57].

## Data Availability

Datasets available upon request from the authors.

## Abbreviations

The following abbreviations are used in this manuscript:

AH	Anterior hypothalamus
ARC	Arcuate nucleus of the hypothalamus
AVPV	Anteroventral periventricular nucleus
BNST	Bed nucleus of the stria terminalis
DMH	Dorsomedial hypothalamus
Hb	Lateral and medial habenula
HPC	Hippocampus (dorsal CA1 and dentate gyrus)
LH	Lateral hypothalamus
LS	Lateral septum
MeAMG	Medial amygdaloid nucleus
MS	Medial septum
MPOA	Medial preoptic area
NAc	Nucleus accumbens
PFC	Prefrontal cortex
PH	Posterior hypothalamus
PVN	Periventricular hypothalamus
VMH	Ventromedial hypothalamus
